# Analysis of Xyloglucan Endotransglycosylase/Hydrolase (*XTH*) Genes and Diverse Roles of Isoenzymes during Persimmon Fruit Development and Postharvest Softening

**DOI:** 10.1371/journal.pone.0123668

**Published:** 2015-04-07

**Authors:** Ye Han, Qinggang Zhu, Zhengke Zhang, Kun Meng, Yali Hou, Qiuyan Ban, Jiangtao Suo, Jingping Rao

**Affiliations:** 1 College of Horticulture, Northwest A&F University, Yangling, Shaanxi, P. R. China; 2 College of Food Science and Technology, Hainan University, Haikou, P.R. China; South China Agricultural University, CHINA

## Abstract

Xyloglucan endotransglycosylase/hydrolase (XTH) enzymes have played a role in the remodeling of cell wall hemicelluloses. To investigate the function of *XTHs* in persimmon (*Diospyros kaki* L.) fruit development and postharvest softening, five cDNAs (*DkXTH1* to *DkXTH5*), whose putative proteins contained the conserved DEIDFEFLG motif of XTH, were cloned. Real time quantitative PCR analysis revealed that *DkXTH1*, *DkXTH4*, and *DkXTH5* peaked in immature expanding fruit, and their higher expression was observed along with higher fruit firmness in cold-treated fruit or firmer cultivar fruit during storage. The opposite gene expression patterns were observed in *DkXTH2* and *DkXTH3*, which reached maxima concomitance with pronounced fruit softening. Meanwhile, the xyloglucan endotransglycosylase (XET) enzymes play important roles in both the rapid growth and ripening of persimmon fruit. Furthermore, the recombined *DkXTH1* and *DkXTH2* proteins showed significant XET activity without any detected XEH activity. However, the XET activity of recombined *DkXTH2* protein had a higher affinity for small acceptor molecules than that of recombined *DkXTH1* protein. The former might prefer to participate in cell wall restructuring, and the latter is more inclined to participate in cell wall assembly. Besides, *DKXTH* proteins could function by targeting to the cell wall under regulation of a signal peptide. The data suggested that individual *DKXTHs* could exhibit different patterns of expression, and the encoded products possessed specific enzymatic properties conferring on their respective functions in growth and postharvest softening of persimmon fruit.

## Introduction

Persimmon (*Diospyros kaki* L.) is an important horticultural commodity with high market value because of its favorable flavor, distinct taste and components, and high nutritional value [1,2]. However, the fruit is quite perishable and susceptible to softening after harvest, which leads to deteriorated quality and major economic losses [3,4]. Fruit softening is considerably attributed to the catabolism of cell wall components. During fruit ripening and softening, pectic and hemicellulosic polysaccharides are the predominant cell wall components undergoing depolymerization and solubilization [5]. These processes involve the coordinated and interdependent action of some cell wall hydrolytic enzymes, including polygalacturonase, pectin methylesterase, pectate lyase, β-galactosidase, expansin, and xyloglucan endotransglycosylase/hydrolase (XTH) [5,6,7,8].

Xyloglucan is a major hemicellulose molecule, which can strengthen the rigidity of the cell wall by forming a skeletal network with cellulose fibrils [9]. XTH, an important enzyme involved in xyloglucan metabolism, exhibits xyloglucan endotransglycosylase (XET) and/or xyloglucan endohydrolase (XEH) activities. XET has a dual role in transferring one xyloglucan molecule fragment (the donor) to another (the acceptor), integrating a newly secreted xyloglucan with a preformed wall-bound one, or restructuring one preformed wall-bound xyloglucan with another [10,11]. As for XEH, water is utilized as an acceptor, resulting in hydrolysis of one xyloglucan molecule [10,12,13].

XTHs associated with disassembly of the cellulose–xyloglucan matrix have contributed to fruit postharvest softening, as reported in kiwifruit, apple, cherimoya, strawberry, and tomato fruits [14,15,16,17,18]. Some researchers proposed that XET activity may be critical in maintaining rather than dismantling the structural integrity of the cell wall [19,20,21,22]. Furthermore, XET activity has been demonstrated to promote cell expansion in rapidly growing cells [14,23,24]. In both tomato and apple fruits, XET activity does not only occur parallel with ripening and postharvest softening, but also occurs in various stages of fruit development, which indicates its importance in fruit expansion [25,26,27]. Thus, the enzymes encoded by *XTHs* may be critical in both fruit growing and ripening. In a previous study, we cloned and characterized two full-length *XTH* genes, namely, *DkXTH1* and *DkXTH2*, from persimmon fruit, and their expression profiles were analyzed during hormone treatment [2]. However, persimmon XTHs are encoded by a large gene family. More genes of the *XTH* family should be identified, and their expression patterns during fruit growth and postharvest softening need to be analyzed for ascertaining their respective characteristics and important functions in different life stages.

The present study aimed to investigate the role of *XTHs* in persimmon fruit during development and softening. Based on the studies by Zhu et al. [2], we continued to isolate three other full-length genes of *XTHs* (*DkXTH3–5*) encoding putative XTHs, and the expression patterns of *DkXTH1–5* were analyzed during persimmon fruit development and softening. To explore the diverse roles of isoenzymes, enzymatic characteristics were tested in recombined *DkXTH1* and *DkXTH2* proteins. In addition, the subcellular localization of *DkXTHs* was elucidated, which is important for predicting their functions.

## Materials and Methods

### 2.1 Plant materials and treatments

Developing persimmon fruits (*D*. *kaki* L. cv Fuping Jianshi) were harvested every 10 d until 150 d after full bloom (DAFB) from a commercial orchard in Fuping Country, Shaanxi, China. The selected fruits were transported to the postharvest laboratory at Northwest A&F University within 3 h. Each group at each sampling time contained 45 fruits divided into three replicates for analysis. The largest diameter of each fruit was measured using a Vernier caliper. After evaluation of firmness, fruits were peeled, cut into small pieces, immediately frozen in liquid nitrogen, and stored at −80°C until use.

For postharvest softening analysis, Fuping Jianshi persimmon fruits were harvested at 150 DAFB with 70%–80% of surface yellow coloration. The selected fruits without blemishes were randomized into two experimental groups, and each group was split into three lots (90 fruits per lot), representing three replicates. We packed each replicate of 90 fruits in a perforated polyethylene bag (0.03 mm thickness). The first group of fruits was stored at 25±1°C (FP-25°C) and the other was stored at 0±1°C (FP-0°C) for analysis. Meanwhile, a firmer cultivar Ganmaokui persimmon fruit was harvested at the same maturity and stored at 25±1°C (GMK-25°C). Samples were randomly selected at four-day intervals to evaluate firmness, respiration rate, and ethylene production. For biochemical and molecular analyses, flesh tissues at the same time interval as above were cut into small pieces, immediately frozen in liquid nitrogen, and stored at −80°C until use.

### 2.2 RNA extraction and *DkXTH* cloning

Total RNA was extracted from the flesh tissue of persimmon fruit harvested at 150 DAFB using the hot borate method [28]. Reverse transcription-polymerase chain reaction (RT-PCR) was carried out using extracted total RNA (1.0 μg), oligo d(T)18, and reverse transcriptase M-MLV (RNase H−) (Takara, Dalian, China). The conserved region of persimmon *XTH* was amplified using degenerate primers previously described [2]. Subsequently, 3'- or 5'-RACE PCR was performed using 3'- or 5'-RACE amplification kits (Takara, Dalian, China), and primers used were designed according to the conserved region gained. The conditions for full-length cDNA amplification were as follows: one cycle at 94°C for 3 min; 35 cycles at 94°C for 1 min, 55°C for 1 min, and 72°C for 1.5 min; and a final cycle at 72°C for 10 min. Primers used for full-length cDNA amplification were designed based on the already cloned 3'- and 5'-untranslated region. All expected amplified fragments were inserted into a pMD19-T vector (Takara, Dalian, China) and then sequenced by Gen Script Co., Ltd. (Nanjing). All primer sequences are listed in Table 1.

**Table 1:** Oligonucleotide sequences for primers used in this study.

Gene name	Gene bank accession number	Prime sequences (5’–3’)	Purpose
*DkXTH1*	JN605344	F: ACGCCAAGTTCTGCGACAC	*DkXTH1* RT-qPCR
		R: GGGTATCGCTTCCTGTCG	
		F: CGGGATCCAAAGGCGGCAACTTCTACC	*DkXTH1* recombinant protein expression
		R:CCCAAGCTTCTAGAGGAACCTCGACCT	
		F:GCTCTAGAATGGCATTCATGTCCTTTC	DkXTH1Full
		R:GGGGTACCGAGGAACCTCGACCTCC	
		F:GCTCTAGAATGGCATTCATGTCCTTTC	DkXTH1sp
		R:GGGGTACCGGCGGCCATCATAGAGCT	
		F:GCTCTAGAAAAGGCGGCAACTTCTA	DkXTH1Int
		R:GGGGTACCGAGGAACCTCGACCTCC	
*DkXTH2*	JN605345	F:ACGCCAAGTTCTGCGACAC	*DkXTH2* RT-qPCR
		R: GGGTATCGCTTCCTGTCG	
		F:CGGGATCCGTACCCAGGAAGCCCGTT	*DkXTH2* recombinant protein expression
		R:CCGCTCGAGTTATATGTCTCTGTCTCTTTTGC	
		F:GCTCTAGAATGGCGATGGGTACCCACTT	DkXTH2Full
		R:CCGCTCGAGTATGTCTCTGTCTCTTTTGCACTCG	
*DkXTH3*	JN605346	Outer: CCTTGTAGTAGGCGTAGAATGG	*DkXTH3* 5’RACE
		Inner: CCCGCTCCTATTTCCCAAGA	
		Outer: CGCATGGGAAGGGTGATAGG	*DkXTH3* 3’RACE
		F:ACATTGGCATGGAAGAAC	*DkXTH3* full-length cDNA clone
		R:CCCCTCGACTGCCAAACA	
		F:GGTGATAGGGAGCAAAGG	*DkXTH3* RT-qPCR
		R:GGACCTTCGGGTATGGA	
*DkXTH4*	JN605347	Outer: GAAGGAAGAAGAGCTGGAAGT	*DkXTH4* 5’RACE
		Inner: AGGTTGCTGTGAAGGGTG	
		Outer: TCACCGGAGGAAAGGGCGAC	*DkXTH4* 3’RACE
		F:ACAAACCCATCAACCAACT	*DkXTH4* Full-length cDNA clone
		R:AACTTCAGGGCAAACAGAG	
		F:ACTTCAATGCCCAGACC	*DkXTH4* RT-qPCR
		R:ACAGCCATTTCTTTAGGG	
*DkXTH5*	KC511052	Outer: TCCGCTTTGAAGTTCCTGTA	*DkXTH5* 5’RACE
		Inner: AAGTCTATCTCGTCGTGGGT	
		Outer: TGGACGGCACGCCCATCAGA	*DkXTH5* 3’RACE
		F:CATTGTATCCAGAAGGCAGAGC	*DkXTH5* full-length cDNA clone
		R:CAGCGGATTGCGCTTGAT	
		F:CCGCTGACCTCAACCAA	*DkXTH5* RT-qPCR
		R:TCCCTGCGACGACAGATAG	
*Actin*	AB219402	F:TGCTCTTCCAGCCATCACTCATT	*Actin* RT-qPCR
		R:ATTTCCTTGCTCATCCGGTCAG	

Letters “F” and “R” indicate the forward and reverse primers, respectively.

### 2.3 Bioinformatic analysis

NCBI Blast program (http://www.ncbi.nlm.nih.gov/BLAST) was used for identifying the nucleotide sequence obtained from RT-PCR. NCBI ORF Finder (http://www.ncbi.nlm.nih.gov/gorf/gorf.html) was applied to analyze open reading frames and protein predictions. Peptide Mass (http://us.expasy.org/tools/peptidemass.html) was used to calculate the mass values and theoretical isoelectric point (*p*I) of deduced amino acid sequences. To predict signal peptides, Signal P (http://www.cbs.dtu.dk/services/SigalP/) was used. Alignment and comparison of the amino acid sequences were performed by DNAMAN. It was based on Bootstrap N-J Tree method (1000 bootstrap trials) by MEGA version 5.1 to generate a phylogenetic tree using deduced amino acid sequences of the five persimmon *XTH* genes (*DKXTHs*) and 23 *XTH* homologues from other species. The three-dimensional structures of putative persimmon *XTH* proteins were predicted using Swiss-Model workspace (http://swissmodel.expasy.org).

### 2.4 Subcellular localization

The full *DKXTH1/2* coding sequence (DkXTH1/2Full), *DKXTH1* signal peptide (DkXTH1sp), and *DKXTH1* sequence without signal peptide (DkXTH1Int) were amplified by PCR. The appropriate restriction enzymes were used to excise the amplified fragments, which are underlined in the primers listed in Table 1. They were then ligated into the pBI 221-GFP vector fused with the *GFP* gene in the 3' region. DNA plasmids (5 μg) were applied to bombard onion epidermal cells by a biolistic PDS-1000/He particle delivery system (Bio-Rad, Hercules, CA, USA). All bombarded onion epidermal cells were then incubated on Murashige–Skoog medium for 24 h at 22°C in the dark, and analyzed using a confocal laser scanning microscope (A1R; Nikon, Japan). When indicated, cells were plasmolyzed in 400 mM sucrose for 15 min.

### 2.5 Expression analysis by real time quantitative PCR (RT-qPCR)

First-strand cDNA was synthesized using total RNA extracted from persimmon tissues as described above. RT-qPCR (20 μL) was repeated in triplicate with 1.0 μL of cDNA (300 ng), 0.8 μL of sense primer and antisense primer (10 μM), 7.4 μL of ddH_2_O, and 10 μL of SYBR Premix Ex Taq II (Takara, Dalian, China) using an iCycler iQ5 (Bio-Rad, USA). PCR conditions were initial heating for 3 min at 95°C, followed by 40 cycles of 10 s at 95°C, 30 s at 55°C, and 20 s at 72°C. The fluorescence was measured at the bottom of each annealing step. A cDNA serial dilution was performed to generate the standard curve, and the expression corrected for persimmon *Actin* was calculated using amplification efficiencies. Primers for *DkXTHs* and persimmon *Actin* are summarized in Table 1. Values were calculated via comparative CT (2^–△△CT^) [29], using the gene expression level at the fruit harvest time point as a nominal value of 1.

### 2.6 Analysis of cell wall polysaccharides

Cell walls were extracted from frozen fruit samples as previously described by Cutillasiturralde et al. [30]. The obtained dry matter of cell walls was sequentially extracted for pectic and hemicellulosic polysaccharides using Na_2_CO_3_ (0.05 M) and KOH (1 and 4 M), respectively, as reported in detail by Cutillasiturralde et al. [30]. The phenol–sulfuric acid method [31] and iodine staining method [18] were applied to determine the contents of hemicelluloses and xyloglucan, respectively. The contents of hemicelluloses and xyloglucan were expressed as μg mg^−1^ cell wall dry weight.

### 2.7 Protein extraction and XET assay

Soluble and ionically bound proteins were obtained using the method described by Cutillasiturralde et al. [30] with some modifications. Frozen fruit samples (3 g) were homogenized in 7 mL of 40 mM sodium acetate (pH 5.0), containing 10 mM β-mercaptoethanol, 13 mM CDTA, 1 M NaCl, and 10% polyvinylpolypyrrolidone. The homogenate was incubated in a shaker (ZWYR-2102C, Zhicheng, Shanghai, China) at 120 rpm min^−1^ for 18 h and centrifuged at 16000 ×*g* for 30 min. After filtration through a 0.45 μm PES membrane (Merck Millipore Ltd. Cork, Ireland), the supernatant was concentrated using an ultrafiltration system (Merck Millipore Ltd. Cork, Ireland) equipped with a 10 kDa membrane cassette and then dialyzed against citrate/phosphate buffer (pH 5.5). All extraction procedures were conducted at 4°C. Coomassie Blue G dye-binding was applied to determine the content of extracted proteins [32].

XET activity was analyzed with a colorimetric method as described by Henriksson and Sulova [33,34]. Tamarind xyloglucan (Megazyme, Bray, Ireland) was digested with *Trichoderma reesei* cellulase (Sigma-Aldrich, St. Louis, MO, USA) to acquire XGOs as previously described. XGOs are a mixture of the oligosaccharides XXXG, XLXG, XXLG, and XLLG [33]. The reaction mixture (0.2 mL) contained 0.2 mg of tamarind xyloglucan, 40 μg of XGOs, 300 μg of enzyme, and 50 mM citrate/phosphate buffer (pH 5.5). When citrate/phosphate buffer was used instead of enzymes and XGOs, the samples were regarded as negative control and blank tubes, respectively. After the mixtures were incubated at 37°C for 30 min, the reactions were stopped by adding 1 M HCl (100 μL). Subsequently, 20% Na_2_SO_4_ (800 μL) and I_2_–KI (1% KI and 0.5% I_2_; 200 μL) were added, and each sample was kept in the dark for 30 min. The optical density of the samples was tested at 620 nm against the blank. The net transglycosylating activity (XETA) was expressed in arbitrary units (a.u.) and calculated according to Sulova et al. [34].

The pH–rate profile of proteins was estimated over the pH range of 3–8 by colorimetric assay. The dependence of relative XET activity of proteins on the concentration of added XGOs was measured by colorimetric method using XGOs ranging from 0.01 mg mL^−1^ to 0.20 mg mL^−1^.

### 2.8 XEH assay and the relative viscosity of persimmon xyloglucans

XEH activity was measured by viscometric assay for depolymerizing xyloglucan, and *T*. *reesei* cellulase (Sigma-Aldrich, St. Louis, MO, USA) was used as the control enzyme. Reaction mixtures (300 μL) contained 3% tamarind xyloglucan, 300 μg of enzyme, and 50 mM citrate/phosphate buffer at pH 5.5. After different periods of incubation, solutions were sucked into a 0.2 mL pipette, and the relative viscosity was calculated by the efflux time [35]. The relative viscosity of persimmon xyloglucans was calculated using 5% total persimmon xyloglucans extracted from fruits during development without enzyme incubation.

### 2.9 Fruit firmness, respiration rate, and ethylene production

Fruit firmness was measured with the peel removed at three positions located at 120° intervals. Firmness was tested by a pressure tester (Model FT327; Effegi, Milan, Italy) equipped with a 5 mm diameter probe, and it was expressed in Newtons (N).

The respiration rate and ethylene production were measured by enclosing six fruits in a 9.17 L vessel for 1 h at storage temperature, and a gas sample (1 mL) was collected using a syringe. A GC-14A gas chromatograph (Shimadzu, Kyoto, Japan) was applied to determine the ethylene concentration as described by Zhu et al. [2]. To measure the respiration rate, CO_2_ levels were analyzed using a CO_2_ infrared gas analyzer (TEL7001, GE Telaire, CA, USA). The fruits were kept with the infrared gas analyzer in a sealable vessel, and CO_2_ production was calculated at 20 min intervals. The respiration rate and ethylene production were expressed as μg kg^−1^ h^−1^ and μL kg^−1^ h^−1^, respectively.

### 2.10 Production and purification of recombinant *XTH* proteins

The mature coding region of *DkXTH1* and *DkXTH2* without the N-terminal leader sequence was obtained by PCR. Products were excised with appropriate restriction enzymes underlined in the primers listed in Table 1 (*BamH*I and *Hind*III for *DkXTH1*; *BamH*I and *Xho*I for *DkXTH2*). Both products were ligated into the pET32a(+) vector (Novagen, USA) and then transformed to *Escherichia coli* BL21 by the heat shock method. Isopropyl β-D-thiogalactopyranoside (0.5 mM) was applied to induce the production of recombinant proteins.

Bacterial cells were lysed by sonication, and the crude proteins were prepared by centrifugation (18000 ×*g*, 10 min). The pellet was dissolved in buffer A (8 M urea, 10% glycerol, 0.5 M NaCl, and 40 mM Tris-HCl, pH 7.9) and filtered through a 0.45 μm PES membrane (Merck Millipore Ltd. Cork, Ireland). The recombinant protein was then purified on a 5 mL Ni-NTA resin column (DP101, TransGen Biotech, Beijing, China) under denaturing conditions. The resin column was equilibrated with buffer A added with 20 mM imidazole, and unbound proteins were washed away using buffer A added with 40 mM imidazole (20 mL). Bound proteins were subsequently eluted using buffer A added with 120 mM imidazole (10 mL). Eluted proteins were diluted to 0.05 mg mL^−1^, and a linear 6 M to 0 M urea gradient in buffer B (6–0 M urea, 0.5 M NaCl, 10% glycerol, 0.3 M L-arginine, 2 mM GSH/0.2 mM GSSG, 1 mM EDTA, and 40 mM Tris-HCl, pH 7.9) was applied to refold eluted proteins by a dialysis bag (MWCO 8–14 kDa, Solarbio, Beijing, China). The refolded protein obtained was concentrated using an ultrafiltration system (Merck Millipore Ltd. Cork, Ireland) equipped with a 10 kDa membrane cassette and then dialyzed against citrate/phosphate buffer (pH 5.5). Purified recombinant proteins were used to analyze enzymatic characteristics as described in Sections 2.7 and 2.8. The empty vector pET32a(+) was used as the blank control. Some purification steps were tested on SDS-PAGE gels stained with Coomassie Brilliant Blue R-250. When mentioned, DkXTH1-RP and DkXTH2-RP refer to purified recombinant *DkXTH1* and *DkXTH2* proteins, respectively.

### 2.11 Statistical analysis

ANOVA was conducted using SPSS version 22.0. Fisher’s least significant difference (LSD) test was used, and P-values below 0.05 were regarded statistically significant (*P* <0.05). Data were plotted on figures as the mean ± standard error of the mean.

## Results

### 3.1. Cloning and phylogenetic analysis of persimmon *XTH* genes

Three full-length sequences, designated *DkXTH3–5*, were isolated from persimmon fruit and all genes were deposited in GenBank with the accession numbers shown in Table 1, column 2. Sequence analysis of *DkXTHs*, including previously obtained *DkXTH1* and *DkXTH2* [2], are summarized in Table 2. The length of *DkXTHs* ranged between 1082 and 1244 bp (Table 2, column 2), and the encoded proteins were between 287–295 amino acid resides (Table 2, column 3). The molecular weights of encoded proteins similarly ranged from 31.93 kDa to 33.53 kDa (Table 2, column 4). However, the five *p*Is of deduced proteins (Table 2, column 5) differed, with the most basic at 9.28 (*DkXTH1*) and most acidic at 5.36 (*DkXTH5*). Signal peptide sequences were predicted for all full-length sequences, except *DkXTH3*, which was predicted to contain no signal peptides (Table 2, column 6). The homology of the amino acid sequences of each persimmon *XTH* to *DkXTH1* ranged from 47.32% to 79.11% (Table 2, column 7).

**Table 2:** Characteristics of *XTHs* isolated from persimmon.

Gene name	Full length(bp)	ORF(aa)	MW (kDa)	pI	SP (no. of aa)	%ID
*DkXTH1*	1082	287	32.55	9.28	25–26	100.00
*DkXTH2*	1243	295	34.32	8.66	23–24	53.54
*DkXTH3*	1244	293	33.53	6.45	no	47.32
*DkXTH4*	1093	287	32.34	8.94	29–30	79.11
*DkXTH5*	1158	287	31.93	5.36	26–27	66.89

Open reading frames (ORF) and protein predictions analyzed using NCBI ORF Finder were given in column 3. The molecular weight (MW, column 4) and isoelectric point (*p*I, column 5) of each mature peptide were calculated by the Peptide Mass programme. The predicted number of amino acids (no. of aa) in each signal peptide (SP, column 6) was predicted using Signal P programme. The percentage amino acid identity (% ID) of each full-length persimmon *XTH* to *DkXTH1* was given in column 7.

**Fig 1:**
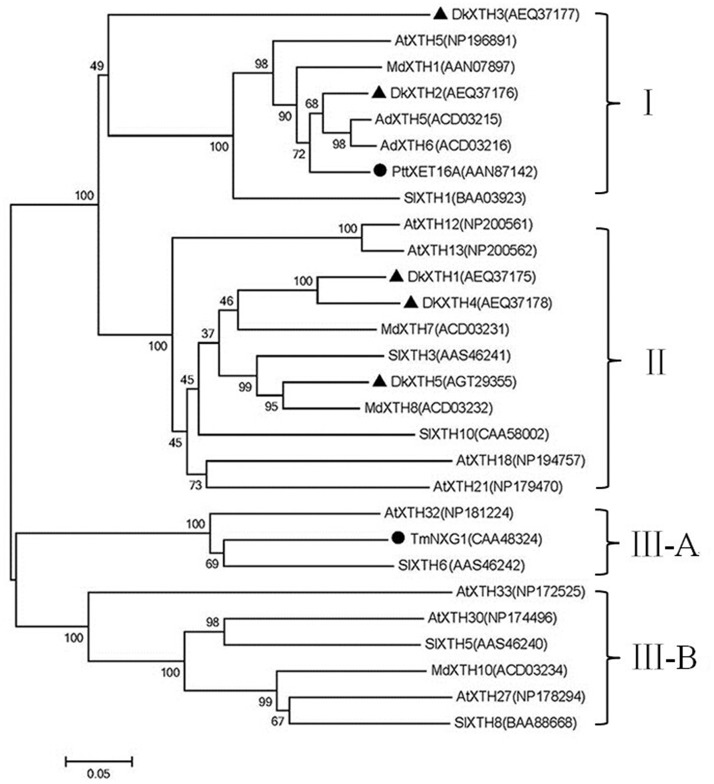
Phylogenetic analysis of deduced amino acid sequences of *XTHs*. Five *DkXTHs* (triangle) and 23 *XTH* homologues from other species were used to generate the phylogenetic tree by MEGA version 5.1 with Bootstrap N-J Tree method (1000 bootstrap trials). Distance scale length of the tree was 0.05. *PttXET16A* and *TmNXG1* (rotundity) were the first XET and XEH with three-dimensional structures, respectively. I, II, III-A, and III-B denote different clades of *XTHs*. GenBank accession numbers are indicated in the figure.

To generate a phylogenetic tree, predicted proteins of the five *DkXTHs* and 23 other *XTH* homologues were used (Fig 1). Phylogenetic analysis revealed that the tree was divided into three major groups, as previously reported [36]. The five persimmon *XTH* putative proteins were placed into divergent groups. *DkXTH2* and *DkXTH3* were classified in group I, along with *AtXTH5*, *MdXTH1*, *AdXTH5*, *AdXTH6*, and *SlXTH1*. Notably, they were grouped with *PttXET16A*, the first XET reported with three dimensional structures [37]. By contrast, *DkXTH1*, *DkXTH4*, and *DkXTH5* were classified in group II, along with *AtXTH18*, *AtXTH21*, *MdXTH7*, *SlXTH10*, *SlXTH3*, *AtXTH3*, *MdXTH8*, *AtXTH12*, and *AtXTH13*. Group III was divided into two groups (III-A and III-B), including *TmNXG1*, which was the first XEH with a three-dimensional structure [38] placed under group III-A.

**Fig 2:**
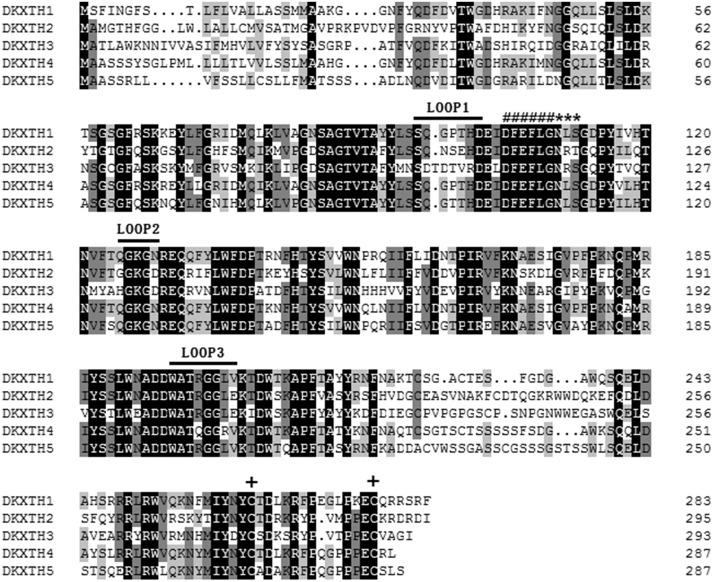
Alignment of the five predicted persimmon *XTH* proteins. Black shading represents identical amino acids, and grey shading identifies the residues shared by at least three of the XTHs. Gaps are indicated by dots to optimize the alignment. Putative catalytic domain, N-glycosylation site, and two cysteines are marked with “#,” “*,” and “+,” respectively. Straight lines identify loops 1–3 of *DkXTH1* and *DkXTH2*. DNAMAN program was used to construct multiple alignments.

Multiple alignment analysis of five persimmon *XTH* putative proteins exhibited a high level of amino acid conservation (Fig 2). All *DkXTHs* contained the conserved DEIDFEFLG motif identified as the catalytic domain of XTH. Beside this conserved motif, a potential N-linked glycosylation (N-X-S/T) site was present and two cysteine residues were found in the carboxyl-terminal region, suggesting that these five *DkXTHs* contained common features with other *XTHs* gained from other plants.

The predicted three-dimensional structures of *DkXTH1* and *DkXTH2* based on the template of the crystal structure of *PttXET16A* (PDB: 1un1) using Swiss-Model workspace are illustrated in S1 Fig Similar to *PttXET16A*, both *DkXTH1* and *DkXTH2* proteins contained three strictly conserved amino acids, which were important in forming the catalytic domain of XTH. Similarly, they contained two disulfide bonds to stabilize the helical extension at the C terminus [36,39]. Three loops were evidently different between *TmNXG1* (PDB: 2uwa) and *PttXET16A* [38]. These loops were loop 1, Asn-84 to Asp-93; loop 2, Glu-117 to Gly-126; and loop 3, Trp-190 to Tyr-197 (in *TmNXG1*). In particular, the length of loop 2 was important in balancing XET and XEH activity [10,38]. When structures of *DkXTH1* and *DkXTH2* were tested, loop 2 had five amino acids equal to *PttXET16A* and less than *TmNXG1* with 10 amino acids (S1 Fig). In detail, loop 2 of *DkXTH1* was formed by amino acids from Gln-125 to Asn-129, and loop 2 of *DkXTH2* was from Gly-131 to Asp-135 (Fig 2).

### 3.2 Subcellular localization

**Fig 3:**
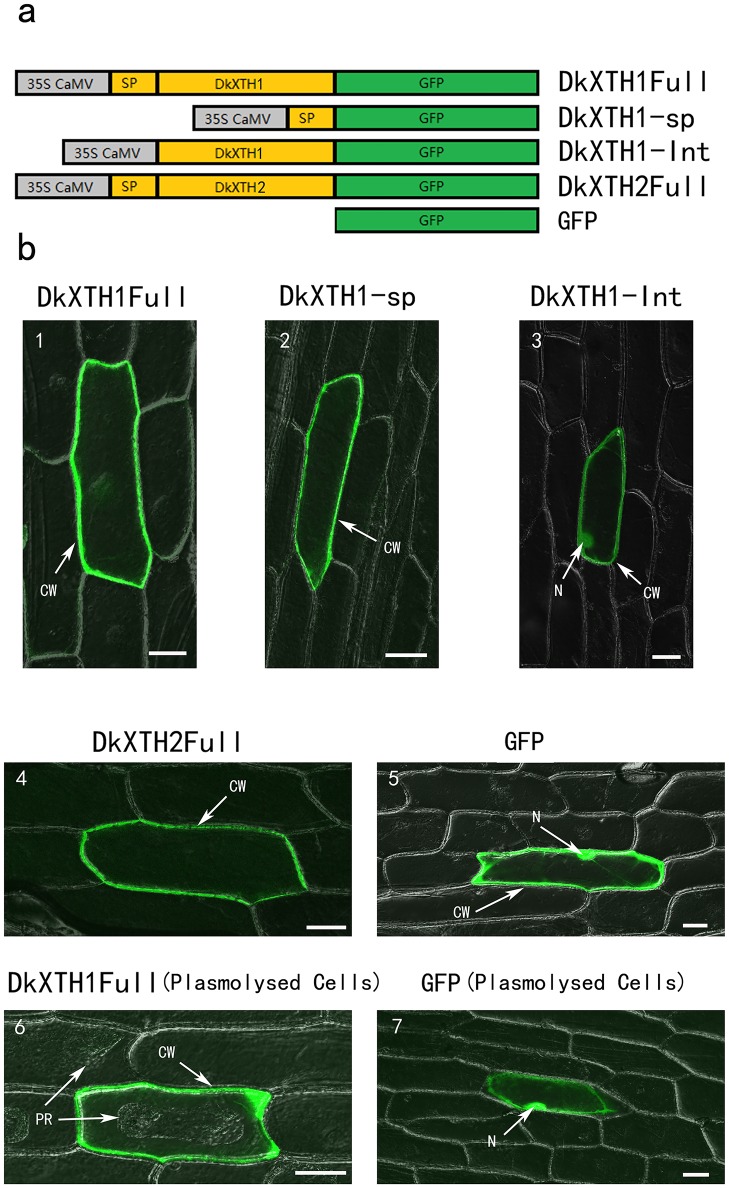
Subcellular localization of *DkXTH1* and *DkXTH2* by transient expression in onion epidermal cells. (a) Diagram of DkXTH1 and DkXTH2 constructs fused to GFP. (b) Panels 1, 2, 3, 4, and 5, transmission and fluorescence images of non-plasmolysed cells; panels 6 and 7, transmission and fluorescence images of plasmolysed cells. Plasmolysis was induced by 400 mM sucrose. CW, cell wall; N, nucleus; PR, protoplast. Scale bar = 50 μm.

To elucidate the subcellular localization of *DkXTH1* and *DkXTH2*, four different constructs fused to GFP (DkXTH1Full, DkXTH1sp, DkXTH1Int, and DKXTH2Full) were transformed to onion cells (Fig 3a). After incubating samples for 24 h, the full-length *DkXTH1* fusion protein (DkXTH1Full) was detected in cell walls of both plasmolyzed and non-plasmolyzed cells (Fig 3b). Similar results were obtained when only the *DkXTH1* signal peptide was fused to GFP (DkXTH1sp). By contrast, cells transformed with the truncated *DkXTH1* that lacked the signal peptide (DkXTH1Int) showed localization in whole cells, similar to GFP control. Similarly, the full-length *DkXTH2* fusion protein (DkXTH2Full) was located in the cell wall. These results indicated that the coding proteins of *DkXTH1* and *DkXTH2* were targeted to the cell wall by their N-terminal signal peptide.

### 3.3 Physiological characteristics of Fuping Jianshi fruit during fruit development

**Fig 4:**
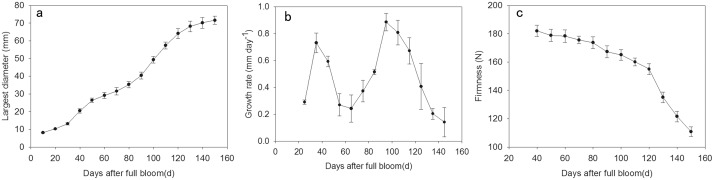
Largest diameter (a), growth rate (b), and firmness (c) during fruit development of Fuping Jianshi. The largest diameter was measured using a Vernier caliper. Each time point was calculated from the means of three biological replicates, and each replicate included 15 fruits, which were from different trees. Vertical bars indicate standard errors of means.

Regarding the increase in size (largest diameter), fruit growth exhibited double sigmoidal kinetics (Fig 4). In detail, fruits at 40 DAFB attained the first maximal growth rate during the first sigmoidal, and fruits at 70 DAFB attained minimal growth rate between the first and second phases. At 100 DAFB, fruits attained the second maximal growth rate during the second sigmoidal and then the growth rate decreased. Meanwhile, fruit firmness was obtained starting at 40 DAFB, and the fruit before that day was too small to measure. During development, fruit firmness decreased from 181.9 N to 110.7 N, and a pronounced softening was observed after 120 DAFB in concomitance with an evident decrease in growth rate.

#### 3.3.1 Cell wall composition

Hemicelluloses were sequentially extracted with 1 and 4 M KOH. As the fruits grew and developed, both hemicellulosic fractions exhibited great quantitative decreases (Table 3). Total hemicelluloses represented 17.97% of cell wall total dry weight at 20 DAFB, which decreased during fruit development to 7.32% at 140 DAFB. The amount of xyloglucan in both hemicellulosic fractions was determined by iodine staining. The amount of xyloglucan decreased during fruit development with a slight increase at 100 DAFB, particularly in 1 M KOH. Subsequently, xyloglucan evidently dropped after 120 DAFB. While, the xyloglucan amount relative to total hemicelluloses was maintained at a high level (30.0%- 43.7%) during fruit development.

**Table 3:** Sugar content of hemicelluloses and xyloglucan (μg mg^−1^ cell wall dry weight) during Fuping Jianshi fruit development.

Days after full bloom(d)	1M KOH Fraction		4M KOH Fraction		Total content		
	Hemicelluloses	Xyloglucan	Hemicelluloses	Xyloglucan	Hemicelluloses	Xyloglucan	Relative Content (%)
20	83.3±6.4^a^	23.5±4.5^a^	96.4±8.0^a^	48.5±5.2^a^	179.7±7.9^a^	72.0±5.3^a^	40.1±3.0^b^
40	75.2±4.9^ab^	18.0±3.3^b^	85.5±4.4^a^	43.1±4.1^ab^	160.7±5.1^b^	61.0±4.1^b^	38.0±2.5^b^
60	65.5±4.7^bc^	10.2±1.3^d^	92.5±3.8^a^	37.1±4.6^b^	157.9±4.7^b^	47.3±3.7^d^	30.0±2.3^c^
80	60.5±5.9^c^	13.0±2.0^bcd^	70.4±5.1^b^	36.9±1.8^b^	130.9±6.0^c^	49.9±2.1^d^	38.1±1.6^b^
100	62.5±3.2^c^	16.3±2.2^bc^	64.4±6.5^b^	38.6±3.6^b^	126.9±5.7^c^	54.9±3.3^c^	43.3±2.6^b^
120	39.2±2.4^d^	11.4±0.8^d^	47.5±4.0^c^	26.4±0.8^d^	86.6±3.6^d^	37.9±0.9^e^	43.7±1.0^a^
140	32.8±3.4^d^	9.0±1.3^d^	40.4±2.6^c^	20.2±2.7^d^	73.2±3.3^d^	29.2±2.3^e^	39.9±3.1^a^

Xyloglucan as a proportion of the hemicellulosic fraction is shown in the last column. Vertical bars indicate standard errors of three replicate assays. Data with different letters at each time point are significantly different (LSD, *P = 0*.*05*).

#### 3.3.2 Relative viscosity of xyloglucan

**Fig 5:**
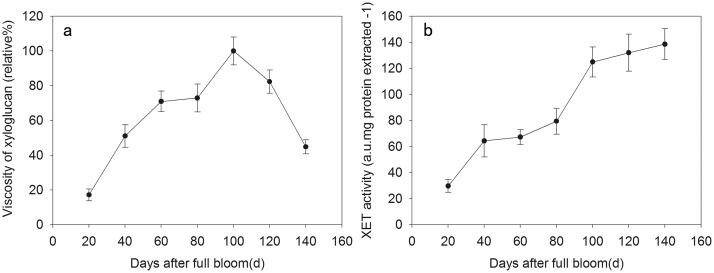
Viscosity of xyloglucan (a) and XET activity (b) during fruit development of Fuping Jianshi. The relative viscosity of xyloglucan was calculated using total xyloglucan extracted with 1 and 4 M KOH from Fuping Jianshi fruit at different stages of development. The XET assay was performed by colorimetric method as described in Section 2.6. Vertical bars indicate standard errors of three biological replicate assays.

The relative viscosity of xyloglucan extracted from persimmon fruit during development was calculated at room temperature. As shown in Fig 5a, the relative viscosity of xyloglucan increased from 20 DAFB to 100 DAFB and then decreased thereafter. Notably, when fruit attained the second maximal growth rate (100 DAFB), the relative viscosity of xyloglucan reached the maximal value. It then rapidly dropped at 120 DAFB, with a pronounced softening.

#### 3.3.3 Changes in XET activity

Proteins extracted from Fuping Jianshi fruits during development were used for measuring XET activity by colorimetric assay. XET activity gradually increased during fruit development, and a pronounced increase occurred at 40 and 100 DAFB, the first and second maximal growth rate stages, respectively. After 100 DAFB, XET activity was maintained at a high level, especially during fruit firmness decreased rapidly (Fig 5b).

### 3.4 Expression of *DkXTHs* during fruit development

**Fig 6:**
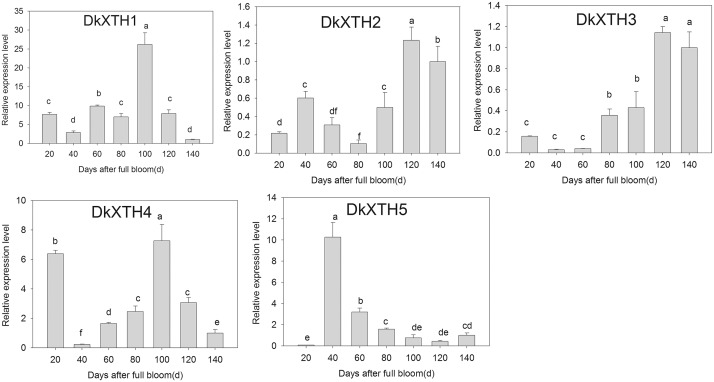
Expression analysis of *DkXTH1-5* during Fuping Jianshi fruit development. Expression level of gene at 140 DAFB was used as the control with a nominal value of 1. Vertical bars indicate the standard error of three biological replicate assays. Columns with different letters at each time point are significantly different (LSD, *P = 0*.*05*).

During fruit development, the expression of five *DkXTHs* was measured by qPCR. RT-qPCR analysis revealed that the expression of *DkXTH1*, *DkXTH4*, and *DkXTH5* was very high in immature growing fruit and peaked before the mature stage (Fig 6). In detail, the expression of *DkXTH5* peaked at 40 DAFB, and that of *DkXTH1* occurred at 100 DAFB, which coincided with the first and second maximal growth rates, respectively. Likewise, *DkXTH4* expression showed a maximum value at both 20 and 100 DAFB. Thereafter, the expression of all these three genes was detected in low levels during the mature stage. However, the expression profiles for *DkXTH2* and *DkXTH3* indicated the contrary pattern with high expression levels at the mature stage. The expression levels of both genes generally exhibited low values in the early stages of fruit growth, but significantly increased during the mature stage and peaked at 120 DAFB with a rapid decline in firmness.

### 3.5 Physiological characterization during persimmon fruit storage

**Fig 7:**
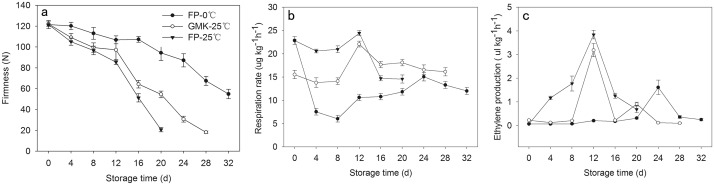
Firmness, respiration rate, and ethylene production of FP-25°C, GMK-25°C, and FP-0°C. FP-25°C indicated Fuping Jianshi fruit stored at 25°C; GMK-25°C indicated Ganmaokui fruit stored at 25°C; FP-0°C indicated Fuping Jianshi fruit stored at 0°C. Vertical bars indicate standard errors of three biological replicate assays.

The firmness of FP-25°C (Fuping Jianshi fruit stored at 25°C) rapidly decreased from 121.5 N to 20.7 N during storage (Fig 7a). FP-0°C (Fuping Jianshi fruit stored at 0°C) showed evident suppression of softening, and the firmness was 20% and 78% higher than that of FP-25°C at 12 and 20 d of storage, respectively. Meanwhile, the firmness of GMK-25°C fruit (Ganmaokui fruit stored at 25°C) showed consistently higher levels than that of FP-25°C fruit, with values 12% and 62% higher at 12 and 20 d of storage, respectively.

The respiration rate exhibited a typical climacteric pattern in all experimental groups during fruit softening (Fig 7b). The maximum respiration rate in FP-0°C (24 d) was only 62% of FP-25°C (12 d), showing the inhibitory effect of low temperature on the respiration rate. The respiration rate in GMK-25°C fruit reached a maximum value at 12 d, but it was slightly lower than that in FP-25°C (*P <0*.*05*).

Ethylene production was suppressed by low temperature treatment, and the maximum ethylene production in FP-0°C (12 d) was only 42% of that in FP-25°C (24 d). In both FP-25°C and GMK-25°C, the peaks of ethylene production were detected at 12 d of storage, but the maximum ethylene production in GMK-25°C was lower than that in FP-25°C fruit (*P <0*.*05*, Fig 7c).

### 3.6 Expression of *DkXTHs* during persimmon fruit storage

**Fig 8:**
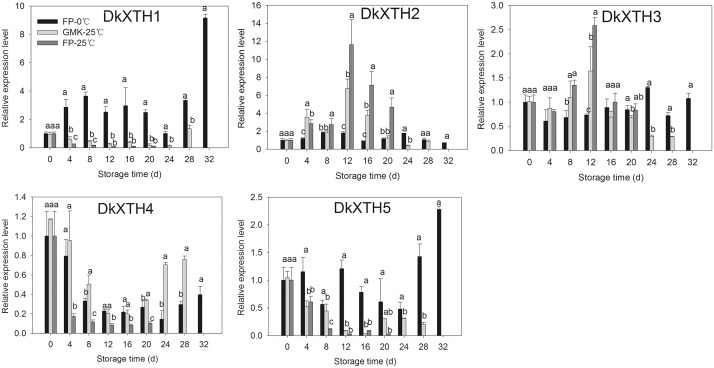
Expression analysis of *DkXTH1-5* in FP-25°C, GMK-25°C, and FP-0°C during storage. FP-25°C indicated Fuping Jianshi fruit stored at 25°C; GMK-25°C indicated Ganmaokui fruit stored at 25°C; FP-0°C indicated Fuping Jianshi fruit stored at 0°C. Expression of gene at 0 d was used as the control with a nominal value of 1. Vertical bars indicate the standard error of three biological replicate assays. Columns with different letters at each time point are significantly different (LSD, *P = 0*.*05*).

After harvesting, the expression of *DkXTHs* in FP-25°C, FP-0°C, and GMK-25°C during storage were also tested by RT-qPCR (Fig 8). In FP-25°C, the expression levels of *DkXTH1*, *DkXTH4*, and *DkXTH5* were lower throughout storage than those at harvest time (0 d). In GMK-25°C, these three genes showed similar expression patterns, but with higher levels at a certain storage time. By contrast, the expression of *DkXTH2* and *DkXTH3* rapidly increased in both FP-25°C and GMK-25°C after harvest, and reached maximum values at 12 d of storage, which coincided with the peak in ethylene production and rapid decline in firmness. Afterward, the expression levels of these two genes steadily declined until the end of storage. The expression levels of *DkXTH1*, *DkXTH4*, and *DkXTH5* were evidently higher in FP-0°C than those in FP-25°C fruit during storage, and low temperature treatment significantly induced the expression of these three genes, especially at the end of storage. However, the expression levels of *DkXTH2* and *DkXTH3* were evidently lower than those in FP-25°C and showed comparatively low values throughout storage.

### 3.7 Recombinant XTH protein expression and activity

**Fig 9:**
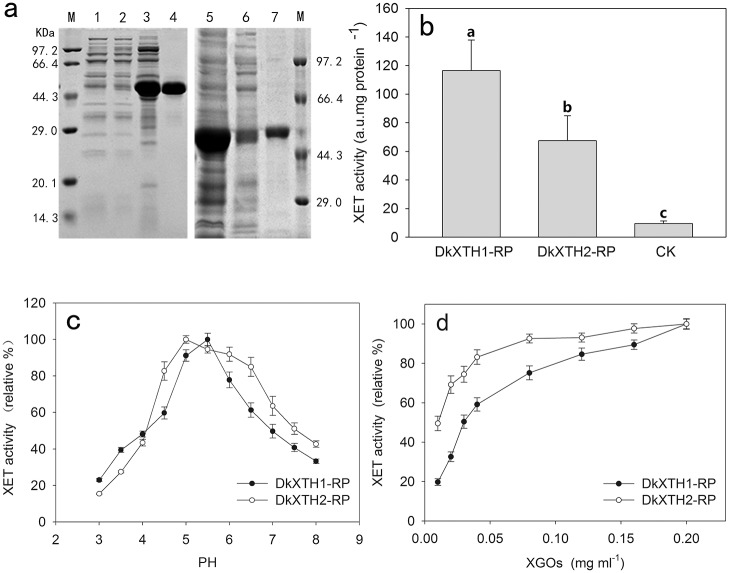
Expression and activity of recombinant XTH proteins. (a) Proteins were separated on SDS–polyacrylamide gels and stained with Coomassie Blue. Lane 1, pET32a(+) control protein; lane 2, unbound protein (*DkXTH1*); lane 3, total protein (*DkXTH1*); lane 4, purified protein (*DkXTH1*); lane 5, total protein(*DkXTH2*); lane 6, unbound protein (*DkXTH2*); lane 7, purified protein (*DkXTH2*); and M, protein marks (Takara, Dalian, China). (b) In vitro XET assay of recombinant *XTH* proteins. The XET assay was performed by colorimetric method as described in Section 2.7. The empty vector pET32a(+) was used as the control. Columns with different letters are significantly different (LSD, *P = 0*.*05*) (c) The pH–rate profile of recombinant *XTH* proteins. (d) Dependence of XET activity of proteins on the concentration of XGOs. Vertical bars indicate standard errors of three replicates.

Two persimmon *XTH* genes *DkXTH1* and *DkXTH2* were expressed in *E*. *coli* and recombinant *XTH* proteins (DkXTH1-RP and DkXTH2-RP) with expected molecular mass were mainly found in the insoluble fraction. The His^6^-tagged proteins were purified using a Ni-NTA resin column under denaturing conditions, and the eluted proteins exhibited high purity (Fig 9a). The eluted proteins were refolded via a reverse urea gradient, and XET activity was measured by colorimetric assay. In contrast to the blank control proteins gained from empty vector-containing bacterial cells, both DkXTH1-RP and DkXTH2-RP showed significant XET activity (Fig 9b), suggesting that active enzymes were produced. Meanwhile, XEH activity was tested by viscometric assay for depolymerizing xyloglucan, and no hydrolytic activity was detected in both DkXTH1-RP and DkXTH2-RP. *T*. *reesei* cellulose, the control enzyme, showed an evident decrease in viscosity of xyloglucan by hydrolysis activity (data not shown).

As shown in Fig 9c, both DkXTH1-RP and DkXTH2-RP exhibited a bell-shaped pH profile, and a rapid loss of activity occurred when the pH ranged from 4 to 5, which is typical for XET isoenzymes [40]. However, DkXTH2-RP exhibited a rather broad optimum pH interval between pH 4.5 and 6.5, whereas DkXTH1-RP was most active for pH values between 5 and 5.5.

The dependence of relative XET activity of DkXTH1-RP and DkXTH2-RP on the concentration of added XGOs was measured by colorimetry (Fig 9d). The relative XET activity of all proteins slightly decreased until the concentrations of XGOs were reduced to 0.04 mg mL^−1^; thereafter, it rapidly dropped at lower oligosaccharide concentrations. The relative XET activity of DkXTH1-RP showed significantly lower values compared with that of DkXTH2-RP when the concentrations of XGOs were below 0.04 mg mL^−1^. When the concentration of XGOs was 0.01 mg mL^−1^, the relative XET activity of DkXTH2-RP was 49.5%, which was significantly higher than that of DkXTH1-RP with 19.7% (*P <0*.*05*).

## Discussion

To date, the roles of XTHs encoded by a large gene family have been comprehensively investigated in a number of fruits, including apple, tomato, kiwifruit, strawberry, and persimmon [2,14,15,18,41,42]. Based on our previous study, in which two full-length *XTH* genes named *DkXTH1* (1082 bp, GenBank accession No.JN605344) and *DkXTH2* (1243 bp, GenBank accession No. JN605345) were cloned in persimmon fruit [2], we continued to identify three full-length *XTHs* designated as *XTH3–5* in our present study, all of which contained the XTH enzyme conserved sequence of DEIDFEFLG (Fig 2), suggesting that they had XET/XEH activities [38]. Phylogenetic tree analysis showed that all *DkXTH*-encoded proteins belonged to group I/II whose almost all coding products exhibited exclusive XET activity (Fig 1) [10,38]. In terms of three-dimensional structures, the length of loop 2 in *Ptt-XET16-34*, which strictly exhibited XET activity, was shorter than that in *Tm-NXG1*, which exhibited exclusive XEH activity [37,38]. An intermediate enzyme, TmNXG1-△YNIIG, was generated by elongating the length of loop 2 in *Tm-NXG1*, and it showed both XET and XEH activities, thereby suggesting that the length of loop 2 could balance the XET and XEH activities of the protein [10,38]. In our study, loop 2 in the predicted three-dimensional structures for both *DkXTH1* and *DkXTH2* had equal lengths with that in *Ptt-XET16-34*, and all of them were shorter than that in *Tm-NXG1* (S1 Fig). Thus, we predict that the proteins encoded by *DkXTHs* might have XET as the main activity, which was directly demonstrated by testing the activity of recombinant *DkXTH1* and *DkXTH2* proteins (Fig 9).

Generally, cell growth is partially attributed to the elongation of xyloglucan chains by integrating newly synthesized xyloglucans into the cell wall matrix under the action of XET [23,43,44,45,46]. In the present study, we found that XET activity continuously increased during persimmon fruit expansion and remained at a high level during fruit ripening (Fig 5). Similar patterns in XET activity were observed in apple, kiwifruit, and tomato fruit during expansion and ripening [25,27]. Measurement of polysaccharide viscosity is a sensitive test reflecting the cleavage or polymerization of polysaccharide backbones, and xyloglucans with different viscosities indicate different lengths of chains [35]. During persimmon fruit development, the relative viscosity of xyloglucan constantly increased with the expansion in fruit size but rapidly decreased at the last period (after 120 DAFB) when firmness dropped (Figs 4 and 5). Similarly, the maintenance of fruit firmness was associated with the increased molecular mass of xyloglucan in *rin* tomato fruit, and the depolymerization of xyloglucan molecules resulted in tomato fruit ripening [26]. These results suggested that XET enzymes may be involved in both the rapid growth and ripening of fruits, and thus genetic regulation of xyloglucan metabolism during fruit development and ripening appears to be particularly crucial.

In the present study, the expression of the five *DkXTHs* exhibited two distinct patterns during persimmon fruit development and softening. The expression of *DkXTH1*, *DkXTH4*, and *DkXTH5* peaked in immature growing persimmon fruit (Fig 6), indicating that they execute pivotal functions for rapid division and expansion of fruit cells. This expression pattern has also been observed in *SlXTH1*, whose transcript level was demonstrated to control the final fruit size in transgenic tomatoes [43]. By contrast, the expression levels of these genes in fruit were lower throughout storage at 25°C (Fig 8), which indicated that they have minor roles in induction of postharvest softening. In addition, the expression levels of *DkXTH1*, *DkXTH4*, and *DkXTH5* in Fuping Jianshi persimmon fruit were markedly induced by cold treatment, and they were also higher in the firmer persimmon cultivar Ganmaokui compared with those in the softer cultivar (Fuping Jianshi) during storage at 25°C (Fig 8). Cold treatment can effectively inhibit ethylene production and delay fruit softening [22,47,48,49]. Therefore, we speculated a correlation between the higher expression of *DkXTH1*, *DkXTH4*, and *DkXTH5* and higher fruit firmness observed in chilled fruit or firmer cultivar fruit during storage at ambient temperatures. For *DkXTH2* and *DkXTH3*, their expression levels were lower in immature fruit and peaked at a mature stage accompanied with a rapid decline in firmness (Figs 4 and 6), which was similar to the expression pattern of *SlXTH5* and *SlXTH8* in tomato fruit during development [41,50]. After harvest, the expression of *DkXTH2* and *DkXTH3* rapidly increased in parallel with increased ethylene production and respiration during fruit postharvest softening (Figs 7 and 8), thereby indicating that the expression of *DkXTH2* and *DkXTH3* could be responsible for ripening and softening in persimmon fruits.

To investigate the various functions of XET isoenzymes, two persimmon *XTH* genes which belonged to different groups and shown distinct expression pattern, were analyzed for subcellular localization, protein expression and enzyme activity analysis, namely, (1) *DkXTH1* (phylogenetic Clade II), in which more *XTH* transcripts were found in immature growing fruit than that in mature fruit; and (2) *DkXTH2* (phylogenetic Clade I), in which more *XTH* transcripts were found in mature fruit than that in immature growing fruit (Figs 1 and 6). Previous studies confirmed that some recombinant XTH proteins can be expressed in yeast or *E*. *coli* [31,41,51,52,53]. In the present study, both DkXTH1-RP and DkXTH2-RP exhibited significant XET activity via a colorimetric assay, and the decrease in viscosity of xyloglucan by hydrolysis was not detected; these findings suggested that DkXTH1-RP and DkXTH2-RP possessed significant XET activity rather than XEH activity (Fig 9b). Similar results were obtained with several other recombined XTH proteins in kiwifruit, apple, and tomato [14,41]. Furthermore, the relative XET activity of DkXTH2-RP was significantly higher than that of DkXTH1-RP at low XGOs concentrations (Fig 9d), suggesting that XET activity of DkXTH2-RP had a higher affinity for small acceptor molecules than DkXTH1-RP. In cultured rose cells, XETs involved in wall restructuring had a higher affinity for small acceptor molecules than those responsible for wall assembly [24,54]. Steele and Fry [54] also demonstrated that the XET isoenzymes extracted from mung bean, which are involved in restructuring of existing wall material, have a higher affinity for small acceptor molecules than that extracted from cauliflower, which are involved in wall assembly by integrating new xyloglucan into the walls. We speculated that the XET isoenzymes encoded by DkXTH1 and DkXTH2 had different roles in cell wall modification, with the former preferring cell wall assembly and playing an important role in cell wall synthesis during rapid fruit growth. This viewpoint was consistent with the report that cell elongation accompanying fruit growth involves substantial wall synthesis at high levels of XET activity in expanding tomato fruit [41]. Moreover, after harvest, this XET enzyme could be responsible for the maintenance of fruit firmness by integrating new xyloglucan molecules into the cell wall, and their decreases during storage might result in fruit softening. Similarly, Miedes et al. [55] suggested that the role of XET in tomato fruit was to maintain the structural integrity of the cell wall, and its decrease during ripening was responsible for fruit softening. DkXTH2-RP preferred to involve in cell wall restructuring, which is necessary for cell wall loosening during fruit ripening and postharvest softening. Notably, the optimum pH values for DkXTH1-RP and DkXTH2-RP differed (Fig 9c). DkXTH1-RP was most active at pH between 5 and 5.5, whereas DkXTH2-RP exhibited a wider range of optimum pH (pH 4.5–6.5). In agreement with our results, several XET isoenzymes isolated from mung (*Phaseolus radiatus* L.) and nasturtium (*Tropaeolum majus* L.), which play different roles in cell wall modification, showed diverse optimum pH values [54,56].

In addition, we observed that both *DkXTH1* and *DkXTH2* were located in the onion cell wall (Fig 3). The recombinant plasmid was still located in the cell wall when only *DKXTH1* signal peptide was used, but the removal of signal peptide resulted in a whole cell location for *DkXTH1*, which suggested that *DKXTH* proteins could play functions by binding to the cell wall under regulation of a signal peptide. Similar results have been observed in maize (*Zea mays* L.), in which *ZmXTH1* is weakly bound to the cell wall [57,58].

In conclusion, the cloned *DkXTHs* played different roles in fruit development and postharvest softening, in which the expression of *DkXTH1*, *DkXTH4*, and *DkXTH5* might be involved in the rapid growth of the fruit and maintenance of fruit firmness during storage, and the expression of *DkXTH2* and *DkXTH3* could be responsible for induction of fruit ripening and postharvest softening. Individual *DKXTHs* exhibited different enzymatic characteristics with distinct patterns of expression, conferring on their respective functions in cell wall modification. In this sense, the encoded XET isoenzymes could play diverse roles in persimmon fruit development and softening.

## Supporting Information

S1 Fig Prediction of three-Dimensional structures of *DkXTH1* and *DkXTH2*. The predicted three-dimensional structures of *DkXTH1* and *DkXTH2* based on the template of the crystal structure of *PttXET16A* using Swiss-Model workspace. (a) Ribbon representation of the three-Dimensional structure of *DkXTH1*. N- to C-terminals are colored from blue to red. The three strictly conserved amino acids E102, D104, and E106 are labeled. (b) Ribbon representation of the three-Dimensional structure of *DkXTH2*. The three strictly conserved amino acids E108, D110, and E112 are labeled. (c) Superimposition of the structures of *DkXTH1* (yellow + blue), *PttXET16A* (gray + magenta), and *TmNXG1* (light blue + red) highlighting the different conformations of three loops. In *TmNXG1*, loop 1 was from Asn-84 to Asp-93; loop 2 was from Glu-117 to Gly-126; and loop 3 was from Trp-190 to Tyr-197. (d) Superimposition of the structures of *DkXTH2* (yellow + blue), *PttXET16A* (gray + magenta), and *TmNXG1* (light blue + red) highlighting the different conformations of three loops.
(TIF)
