# Supplementary figures and images for: Analysis of Xyloglucan Endotransglycosylase/Hydrolase (XTH) Genes and Diverse Roles of Isoenzymes during Persimmon Fruit Development and Postharvest Softening
Source: PLoS One. 2015 Apr 7;10(4):e0123668. doi: 10.1371/journal.pone.0123668 (PMC4388718; doi:10.1371/journal.pone.0123668)

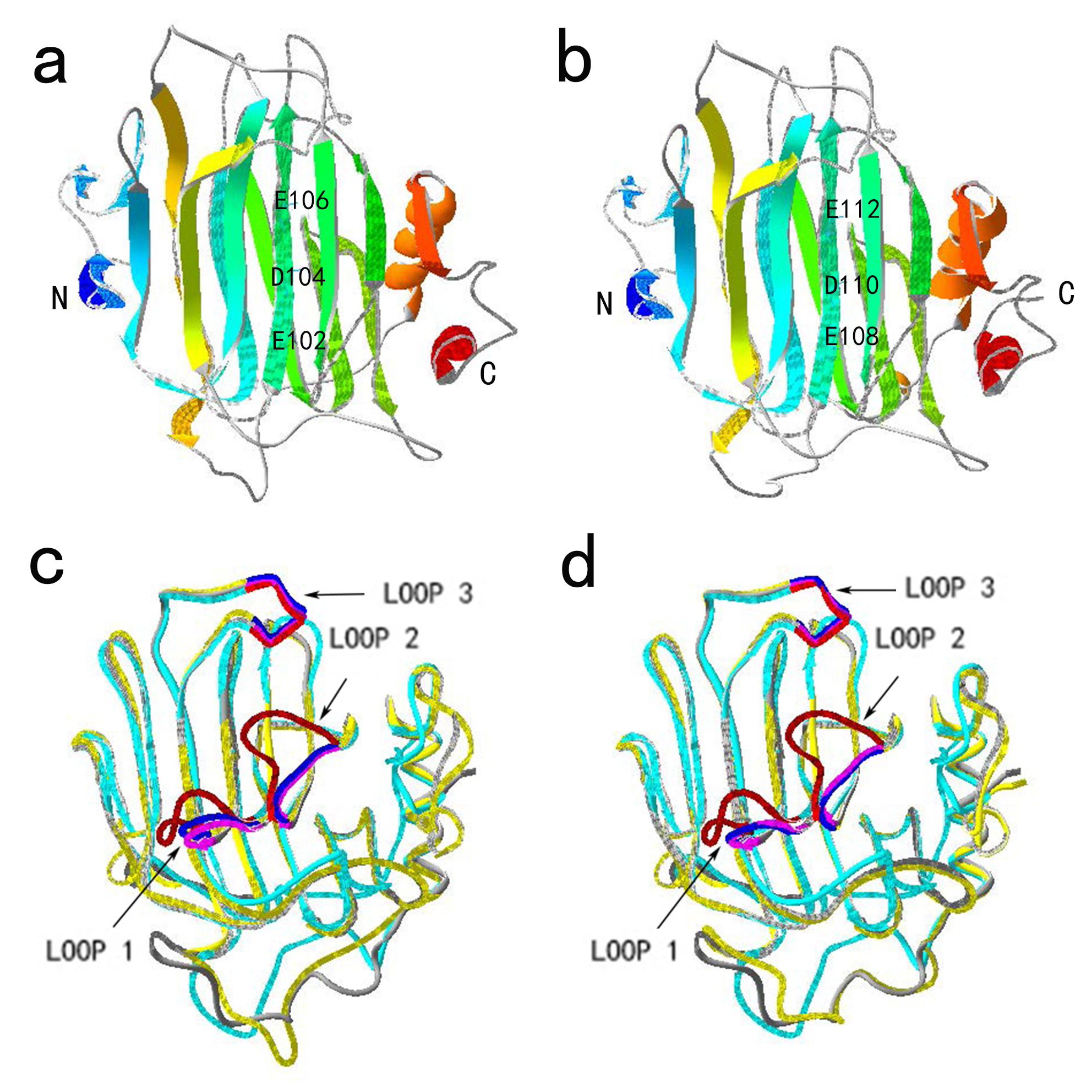

Supplement: S1 Fig — The predicted three-dimensional structures of DkXTH1 and DkXTH2 based on the template of the crystal structure of PttXET16A using Swiss-Model workspace. (a) Ribbon representation of the three-Dimensional structure of DkXTH1. N- to C-terminals are colored from blue to red. The three strictly conserved amino acids E102, D104, and E106 are labeled. (b) Ribbon representation of the three-Dimensional structure of DkXTH2. The three strictly conserved amino acids E108, D110, and E112 are labeled. (c) Superimposition of the structures of DkXTH1 (yellow + blue), PttXET16A (gray + magenta), and TmNXG1 (light blue + red) highlighting the different conformations of three loops. In TmNXG1, loop 1 was from Asn-84 to Asp-93; loop 2 was from Glu-117 to Gly-126; and loop 3 was from Trp-190 to Tyr-197. (d) Superimposition of the structures of DkXTH2 (yellow + blue), PttXET16A (gray + magenta), and TmNXG1 (light blue + red) highlighting the different conformations of three loops. (TIF) [file pone.0123668.s001.tif]
